# Gene Expression-Based Classification of European Seabass Larval Batches According to Saddleback Syndrome Incidence Using Machine Learning

**DOI:** 10.3390/ani16152375

**Published:** 2026-08-03

**Authors:** Andreas Tsipourlianos, Alice Printzi, Alexia Fytsili, Lamprini Tzioga, Soraia Santos, Babak Najafpour, Deborah M. Power, George Koumoundouros, Katerina A. Moutou

**Affiliations:** 1 Department of Biochemistry and Biotechnology, University of Thessaly, Biopolis, 41500 Larissa, Greece; 2 Biology Department, University of Crete, 70013 Heraklion, Greece; 3 Centro de Ciências do Mar, University of the Algarve, 8005-139 Faro, Portugaldpower@ualg.pt (D.M.P.)

**Keywords:** European seabass, *Dicentrarchus labrax*, saddleback syndrome, skeletal deformities, larval quality, gene expression, machine learning, random forest, aquaculture, fish hatcheries

## Abstract

Skeletal deformities remain a major challenge in marine fish hatcheries, affecting larval quality, animal welfare, production efficiency, and market value. In European seabass (*Dicentrarchus labrax*), saddleback syndrome (SBS) is a relevant skeletal abnormality that develops during larval ontogeny and has been associated with defects of the primordial marginal finfold around the flexion stage. This study investigated whether gene expression markers, combined with machine learning, could provide a stage-specific molecular approach for assessing SBS-associated larval batch quality. Larval populations from commercial hatcheries were classified as GOOD or POOR according to SBS incidence at mid-metamorphosis. Gene expression was analyzed at first feeding, flexion, post-flexion, and mid-metamorphosis, targeting genes involved in osteogenesis, myogenesis, metabolism, oxidative phosphorylation, and stress response. Stage-specific random forest models were used to classify gene expression profiles derived from larval populations with contrasting SBS incidence and to identify candidate informative genes. The models showed cross-validated area under the receiver operating characteristic curve (ROC AUC) values ranging from 0.83 to 0.962, with the highest performance at flexion. Reduced models based on the three most informative genes retained comparable internal cross-validation performance. Key candidate genes were mainly related to mitochondrial energy production, iron metabolism, stress response, muscle development, and extracellular matrix formation. These findings suggest that gene expression profiling combined with machine learning may support stage-aware discrimination of larval populations with contrasting SBS incidence, although validation in larger independent datasets is required before hatchery application.

## Simple Summary

Skeletal deformities reduce larval quality, animal welfare, production efficiency, and market value in marine fish hatcheries. This study investigated whether gene expression could help assess the likelihood of saddleback syndrome (SBS) in larval batches. SBS is a skeletal abnormality linked to defects in the primordial marginal finfold around the flexion stage. Larval batches were classified as GOOD or POOR according to saddleback syndrome incidence at mid-metamorphosis. Gene expression was determined at first feeding, flexion, post-flexion, and mid-metamorphosis, and stage-specific random forest models were used to classify batch SBS incidence. The models showed the strongest performance at flexion, consistent with the developmental period previously associated with saddleback syndrome onset. The most informative genes were mainly related to mitochondrial energy production, iron metabolism, stress response, muscle development, and extracellular matrix formation. These findings identify gene expression signatures associated with SBS incidence and suggest that gene expression profiling combined with machine learning may support stage-aware assessment of SBS-associated larval quality, although larger independent datasets are required before hatchery application.

## 1. Introduction

The commercial success of fish farming depends on the availability of high-quality juveniles for on-growing. Optimizing rearing protocols, considering both biotic and abiotic parameters, including larval density, feeding protocols, and health care, is essential for efficient larval development. Skeletal abnormalities provide a significant challenge since they impede fish normal development, resulting in postponed growth, increased mortality rates, and poorer overall animal welfare [1,2,3]. Skeletal abnormalities have a negative impact on fish swimming ability, feed conversion rate, growth rate, survival, stress resistance, and susceptibility to pathogens and bacteria, as thoroughly reviewed by Boglione and colleagues [4]. Deformed fish require manual culling, thus raising labor costs, while an abnormal fish shape erodes consumer confidence in aquaculture products [5], ultimately diminishing their market value and threatening the industry’s sustainability. The phenotypic manifestation of skeletal abnormalities is diverse and can range from mild abnormalities, affecting a few internal skeletal elements, to more severe abnormalities that have a noticeable impact on the fish phenotype.

Skeletal anomalies in most aquaculture species emerge mostly during the embryonic and larval stages [4] and constitute a key performance indicator (KPI) for hatcheries [2]. In commercial hatcheries, the average incidence of such deformities can reach approximately 20%, with certain production batches or cycles exhibiting substantially higher rates, occasionally escalating to over 45% [4]. Skeletal abnormalities are predominantly attributed to a combination of abiotic [6,7,8,9], nutritional [10,11,12], and genetic factors [9,13,14]. Additional contributing influences may include infectious or disease-related conditions [15], maternal effects associated with egg quality [16], and epigenetic mechanisms [17,18].

The partial or complete absence of the dorsal fin, known as saddleback syndrome (SBS), represents one of the most extensively studied fin deformities in cultured fish. This abnormality has been documented in association with the underdevelopment of other fins, including the paired fins in blue tilapia (*Sarotherodon aureus*) [19], the caudal fin in common dentex (*Dentex dentex*) [20], and the anal and pelvic fins in European seabass (*Dicentrarchus labrax*) [21]. In farmed fish, SBS typically arises during the larval stage [20,21,22]. In larval *Dicentrarchus labrax*, SBS has been observed at frequencies exceeding 65% in certain cases, with its ontogenetic origins traced to early abnormalities of the primordial marginal finfold during the flexion stage [21]. This ontogenetic sequence implies that molecular markers sampled before, during, or after flexion are unlikely to have equivalent biological meaning: pre-flexion signatures may reflect larval susceptibility, flexion-stage signatures may be associated with ongoing pathogenic processes, whereas later signatures may reflect progression or consequences of an already developing phenotype.

Regular fish quality monitoring is widely acknowledged as being essential in modern aquaculture to support the early identification of emerging rearing issues or unintentional deviations from the complex hatching protocols in order to apply mitigation strategies [2]. Monitoring for skeletal abnormalities primarily relies on two methods: the double-staining protocol (alcian-blue and alizarin-red S) and X-ray analysis. Both methods require significant technical effort and time; nevertheless, they are crucial for identifying and manually culling deformed fish from the production cycle as early as possible to avoid wasting resources on suboptimal growth [4].

In recent decades, there has been a growing body of research investigating the molecular mechanisms underlying skeletal deformities in fish, driven by increasing concerns about farmed fish quality, animal welfare, and production sustainability. Gene expression, as well as transcriptomic and proteomic studies, have revealed altered gene expression related to bone development and mineral metabolism in species such as Atlantic salmon (*Salmo salar*) [23,24], Murray cod (*Maccullochella peelii*) [25], largemouth bass (*Micropterus salmoides*) [26], and rainbow trout (*Oncorhynchus mykiss*) [27]. Nutritional and environmental factors, like vitamin D3/K3 interactions [28], dietary phospholipid deficiency [26], hyperthermia [23], and endocrine disruptors [29], have been shown to disrupt bone-related gene networks. In common carp (*Cyprinus carpio*), spinal deformities are associated with genes regulating nervous system development and growth [30]. Genetic, gene expression, and histological approaches have also identified candidate genes linked to specific anomalies such as mandibular prognathism, caudal-fin abnormalities, and ray resorption syndrome in European seabass [3,14,31]. While studies such as these significantly advance our understanding of the molecular and environmental factors driving altered skeletogenesis, their translation into practical tools remains limited. As highlighted by Chandra and colleagues [15], deformities continue to pose major biological, ethical, and economic challenges in aquaculture, in part due to persistent gaps in our understanding of the underlying mechanisms and the absence of early-stage, low-cost diagnostic tools. Although molecular markers hold potential for identifying deformity-associated developmental trajectories during embryonic and larval development, reliable stage-specific predictors have yet to be established. Bridging this gap requires targeted efforts to harness current molecular insights toward the future development of approaches for early detection and prevention, ultimately improving fish quality and sustainability in aquaculture systems.

Nowadays, artificial intelligence (AI) and machine learning (ML) have been employed in aquaculture in the form of automatic or semi-automatic computer-vision-based image processing techniques due to their high detection accuracy in identifying fish pathologies. AI and ML are currently being used to refine feeding practices, monitor water quality [32], and enhance the overall health and yield of fish stocks [33,34], as recently reviewed by Mandal & Ghosh [35]. Artificial intelligence and machine learning are already applied in the analysis of skeletal deformities in aquaculture, but their use is mainly based on image analysis [36,37,38]. While this application is promising and increasingly adopted, it is still limited by (1) the significant technical effort and time that is required to produce the primary data (sample preparation), (2) how early in ontogeny is the application time window, and (3) limitations of the methodology itself, such as variations in lighting, pose, background, and water turbidity [39], leading to a decrease in the performance of the models. Moreover, monitoring skeletal deformities by image analysis relies on the visible manifestation of the phenotype, whereas molecular methods may help detect batch-level susceptibility or deformity-associated developmental trajectories before routine phenotypic screening becomes informative. Gene expression profiling was selected because it provides a direct and experimentally accessible measure of active molecular responses across developmental stages and can be applied to targeted candidate pathways using established quantitative PCR methods. Metabolomic and epigenetic approaches could provide complementary information in future investigations.

In this study, we employed a diverse collection of European seabass (*Dicentrarchus labrax*) larval samples originating from two commercial hatcheries. Larval batches were classified as “GOOD” or “POOR” according to SBS incidence at mid-metamorphosis, while gene expression was analyzed across four preceding developmental stages: first feeding, flexion, post-flexion, and mid-metamorphosis. We targeted genes involved in skeletal muscle development, osteogenesis, metabolic regulation, oxidative phosphorylation, and stress response, and used these data to train stage-specific random forest models for batch-quality classification. These functional categories were selected to capture molecular processes plausibly associated with the development of the SBS phenotype. As a biological rationale for selecting these categories, abnormal formation of the primordial marginal finfold and fin rays may be associated with changes in extracellular matrix production and skeletogenic and myogenic development, while genes related to energy metabolism and oxidative phosphorylation were included to reflect the energetic demands of rapid larval growth and tissue differentiation. Stress- and metabolism-related genes were also included because the larval physiological condition and environmental stress can influence developmental trajectories and the occurrence of skeletal abnormalities. Given the known ontogenetic origin of SBS around the flexion stage, our aim was not only to evaluate whether gene expression profiles can discriminate batches with divergent SBS outcomes, but also to determine how the predictive and biological relevance of molecular markers changes across development. Specifically, we sought to determine whether early-stage expression profiles reflect pre-onset susceptibility, whether flexion provides a biologically meaningful window for assessing gene expression patterns associated with SBS incidence, and whether later stages mainly capture progression or molecular signatures of an already developing phenotype. This proof-of-concept framework is intended to support the future development of stage-aware molecular screening approaches for hatchery monitoring of SBS incidence.

## 2. Materials and Methods

### 2.1. Sample Origin

From each of the two participating hatcheries, 1–2 populations per production cycle were randomly selected across a full production year (16 populations in total). Larval rearing was performed in accordance with the established protocols for the species. Larvae were reared initially in the presence of background phytoplankton (6–30 days post-hatching, dph), with initial feeding on enriched rotifers (6–24 dph), followed by gradual provision of Artemia instar I (8–21 dph) and enriched instar II nauplii (12–55 dph), and finally of inert commercial diets (>30 dph).

### 2.2. Evaluation of Skeletal Quality Based on SBS Incidence

The occurrence of skeletal abnormalities across the respective larval populations was assessed using specimens collected during the mid-metamorphic stage (39–56 days post-hatch, approximately 12–19 mm standard length, SL). From each population, a random sample of 50 individuals was taken. Specimens were anesthetized using ethylene glycol monophenyl ether (0.2–0.5 mL L^−1^) and subsequently fixed in 5% phosphate-buffered formalin (pH 7.2). Bone and cartilage were stained following the protocol described by Walker and Kimmel [40]. Standard length (SL) was measured post-staining.

### 2.3. Larval Sampling for Gene Expression Analysis

Since SBS develops during the larval period [21], gene expression analysis focused on samples collected from the same populations at four developmental stages during this period: first feeding (FF, 8–9 dph), flexion (FL, 20–22 dph), post-flexion (PF, 31–34 dph), and mid-metamorphosis (MM, 39–56 dph). A total of 142 RNA samples were analyzed, comprising 33 samples at FF, 26 at FL, 41 at PF, and 42 at MM. From each of the two participating hatcheries, four populations were selected based on their skeletal quality evaluation: two populations exhibiting a high incidence of SBS and two with low incidence (Table A1). Samples for RNA extraction were collected in sterile 15 mL or 50 mL tubes containing 10 mL or 30 mL of RNAlater, respectively, depending on the sample volume. All samples were stored in RNAlater at −20 °C until RNA extraction. The phenotypic classification was performed at the larval population level. Gene expression samples were obtained from larvae belonging to the same populations as those used for skeletal evaluation, but not from the same individuals. Multiple independent RNA samples were obtained from each larval population. Each RNA sample was treated as a separate observation and assigned to the GOOD or POOR group according to the SBS classification of its population of origin. Therefore, the analysis evaluates associations between gene expression profiles and population-level SBS incidence and does not predict the SBS status of individual larvae.

### 2.4. Gene Expression Analysis

For RNA extraction, larvae were removed from the RNAlater at 4 °C and weighed. The number of larvae per sample used for the RNA extracts varied with size, and approximately 10–15 larvae were used/sample from FF, 10 larvae were used/sample for FL, 3 larvae were used/sample for PF, and 1 larva was used for MM. Larvae were defrosted in lysis buffer and homogenized by mechanical disruption with two iron beads (5 mm) using a Tissue lyser II (Qiagen, Hilden, Germany) and 3 cycles (30 Hz) of 30 s at room temperature. Total RNA from whole larvae was extracted using an E.Z.N.A. Total RNA Kit I (Omega Bio-Tek, Norcross, GA, USA) according to the manufacturer’s instructions. To remove any residual genomic DNA from the isolated RNA, all samples were treated using a DNase I Digestion Set (Omega Bio-Tek) during column purification, following the manufacturer’s instructions. RNA quality and integrity were initially checked using a Nanodrop spectrophotometer and 1% agarose gel electrophoresis. PCR reactions were performed in a 10 ul reaction volume containing 5 ng RNA. The PCR thermocycle was as follows: 95 °C for 3 min, followed by 25 cycles of 95 °C for 20 s, 58 °C for 20 s, 72 °C for 20 s, and a final extension step of 72 ◦C for 5 min. The amplified PCR products were visualized on a 2% TAE agarose gel. Only RNA that had no amplified 18S rRNA was used for cDNA synthesis. For cDNA synthesis, 500 ng of DNase-treated RNA was used, and cDNA synthesis was performed in 96-well plates using a PrimeScript-RT reagent kit (Takara Bio Inc., Kusatsu, Shiga, Japan), following the manufacturer’s instructions. In brief, a 20 μL final reaction volume contained 100 pmol random hexamers (Jena Bioscience, Jena, Germany), 1 mM dNTPs (Nzytech, Lisboa, Portugal), 200 U of enzyme, and 20 U RNase Inhibitor (Nzytech, Portugal). The success of the cDNA synthesis was verified by PCR using specific primers for the b-actin gene according to the following amplification cycle: 5 min at 95 °C, followed by 35 cycles of 95 °C for 15 s, 60 °C for 20 s, and 72 °C for 20 s, followed by 10 min at 72 °C.

A number of genes representing key biological functions, such as osteogenesis, myogenesis, metabolism regulation, and energy production, were targeted as potential molecular markers. PCR reactions for the European seabass samples of a total of twenty-six genes were analyzed; from those, twenty-four were candidate markers and two housekeeping genes (Table A2). PCR reactions were prepared for a 10 µL final volume using the KAPA SYBR^®^ FAST qPCR Master Mix (2X) Kit (Kapa Biosystems, Wilmington, MA, USA) and the appropriate concentration of each transcript-specific set of primers. Primers were designed using Primer3 (v.0.4.0) [41] and Beacon Designer^TM^ (v8.21) software, and all primers were designed to span the coding sequences of the transcripts. The amplification cycle was as follows: 5 min at 95 °C, followed by 40 cycles of 95 °C for 20 s and 60 °C for 20 s, followed by the dissociation curve step to verify a single-product amplification. Each reaction was performed in technical duplicate, and duplicate measurements were accepted when the difference between Cq values did not exceed 0.5 cycles. A standard curve using a dilution series (1:5, 1:10, 1:20, 1:50, 1:100) from pooled cDNA was prepared to estimate amplification efficiency (Table A2). Two housekeeping genes (*rpl13*, *fau*) were selected using the RefFinder [42] algorithm. From the obtained Ct values, R0 was calculated using the following formula: R0 = *Threshold*/(1 + *efficiency*)*^ct^* and was normalized using the geometric mean of the level of expression of the two housekeeping genes.

### 2.5. Statistical Analysis

For the statistical analysis, a collection of R (version 4.5.1) [43] packages was used. To examine the normality of the distribution for each variable, we used the Shapiro–Wilk test to examine whether the variables were normally distributed and qqplots to examine the degree of skewness of the data. 

**Figure 1:**
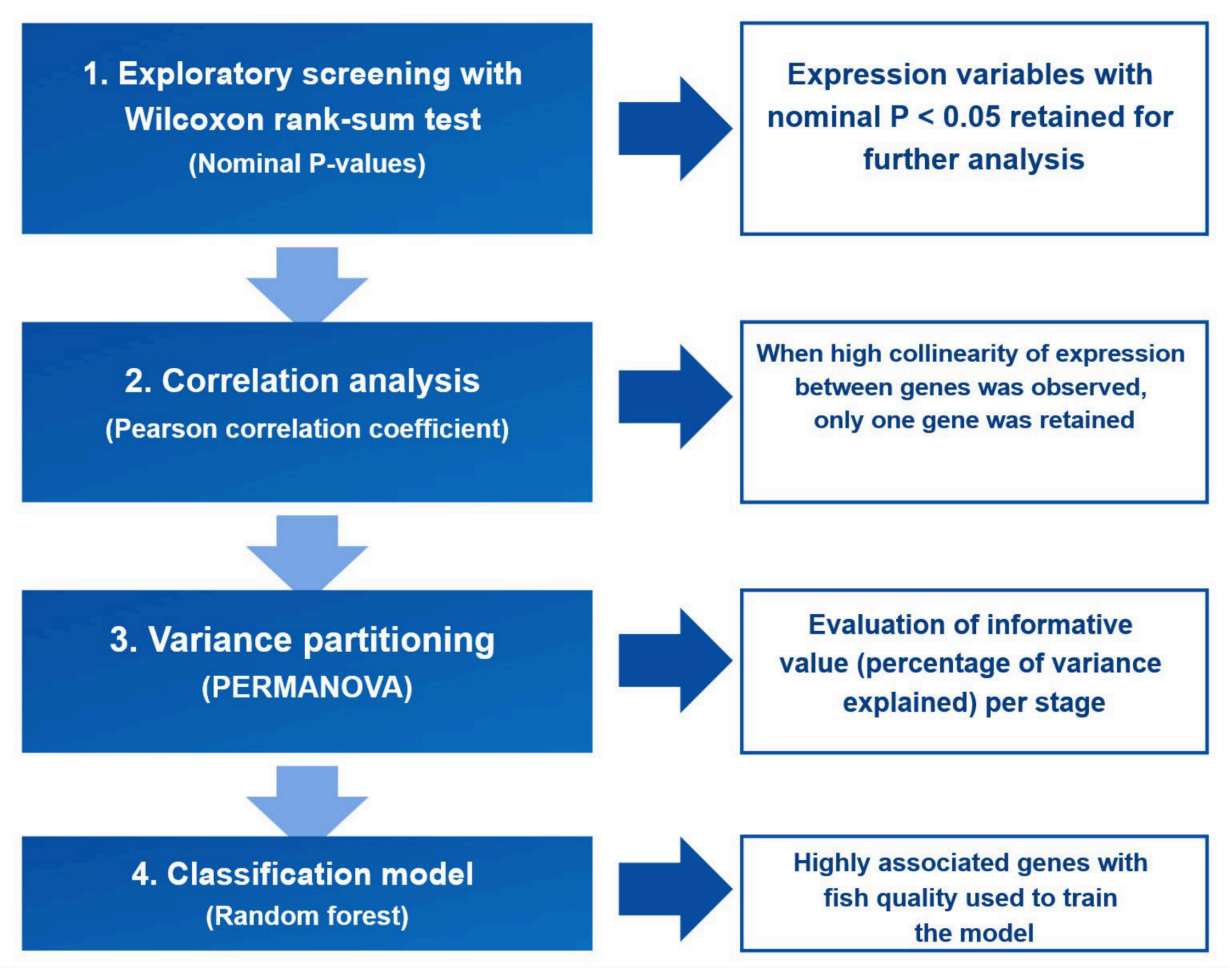
Schematic workflow of statistical analysis.

A structured statistical analysis that included several tests was implemented to identify and retain the most relevant variables for subsequent analyses (Figure 1). The Wilcoxon rank-sum test was used as an exploratory feature-screening step to prioritize expression variables showing the strongest univariate differences between the GOOD and POOR groups at each developmental stage and to reduce the number of variables entered into the random forest models. This filtering strategy was used to limit model complexity and improve interpretability given the relatively small stage-specific sample sizes. It was not intended to imply that genes without nominally significant univariate differences lacked potential multivariate predictive value. Nominal *p*-values were used for this screening purpose rather than as confirmatory evidence of differential expression. Benjamini–Hochberg false-discovery-rate correction was additionally applied separately within each developmental stage across the 25 tested expression variables to assess the robustness of the individual associations. FDR-adjusted *p*-values were used only as a robustness assessment and were not used as a criterion for feature selection. Pearson correlation coefficients were used to identify strong linear redundancy among normalized expression variables before random forest modeling. The coefficients served as a predictor-filtering criterion rather than as tests of biological associations between individual genes. Gene filtering by Wilcoxon testing and correlation analysis was performed once on the complete stage-specific datasets before cross-validation and was not repeated within individual cross-validation folds. To evaluate whether there were differences between the two SBS-based quality groups at each stage, a multivariate approach was followed, and to evaluate the amount of the variability explained by the groupings, we conducted permutational analysis of variance (PERMANOVA) [44]. Prior to that, to be sure that the assumptions of PERMANOVA were met, we conducted a dispersion analysis [44] to examine whether the dispersion of the data was similar between the two groupings.

To classify samples as either “GOOD” or “POOR”, a random forest (RF) [45,46] model was developed using the caret package (version 7.0-1) in R. The stage-specific sample numbers reported in Table A1 were used in the corresponding random forest analyses. Training and validation partitions were generated internally using repeated 10-fold cross-validation, repeated five times; no separate fixed holdout test set was used. Cross-validation folds were generated at the individual RNA-sample level and were not grouped by larval population. Given the imbalance in class distribution, synthetic minority over-sampling (SMOTE) was applied during resampling to improve model generalization across both classes. The model selection metric was the area under the ROC curve (AUC). The random forest mtry parameter was tuned using the repeated cross-validation resamples, with candidate values automatically generated by caret (tuneLength = 5) and the optimal value selected according to ROC AUC. The random forest was configured with 1000 trees and a minimum node size of 5. Variable importance was assessed to identify the most predictive features of SBS-associated batch-quality status while providing further insight into the biological relevance of the findings. For each developmental stage, the reduced three-gene model was constructed using the three predictors with the highest Mean Decrease Accuracy values from the corresponding full random forest model. Internal model performance was evaluated using cross-validated ROC AUC, sensitivity, and specificity, obtained from the repeated cross-validation procedure. Fitted model metrics calculated on the training data were additionally reported descriptively.

## 3. Results

### 3.1. Occurrence and Frequency of Saddleback Syndrome (SBS) in Dicentrarchus labrax Larvae

**Figure 2:**
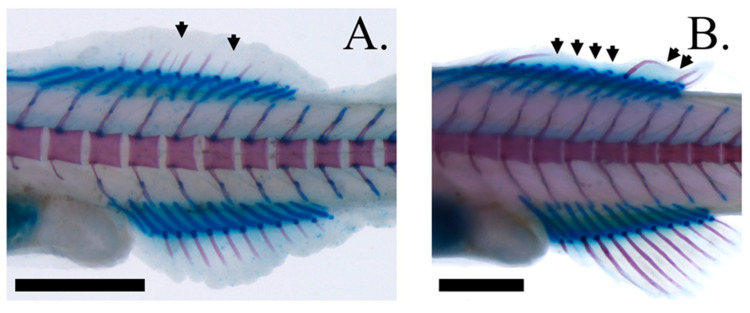
Representative cases of saddleback syndrome (SBS) in the samples examined. (**A**). Abnormal development of fin rays (arrows), associated with a thickened and damaged primordial marginal finfold. (**B**). Absence of fin rays (arrows). Scale bars = 1 mm.

Saddleback syndrome (SBS) was observed in all examined samples, characterized by the typical phenotype of a thickened and damaged primordial marginal finfold (Figure 2A), as well as missing (Figure 2B, arrows) or abnormally developing fin rays (Figure 2A, arrows). The frequency of SBS within samples ranged from 2% to 66%, with a mean of 28.0 ± 16.9% (±SD). Four populations with SBS frequencies ranging from 2% to 18% (mean ± SD: 9.5 ± 6.6%) were classified as of GOOD quality, while four populations with SBS frequencies between 36% and 66% (mean ± SD: 49.0 ± 12.5%) were classified as of POOR quality. Remaining skeletal abnormalities that were present in the samples examined consisted of lower-jaw elongation (0–6%) and vertebral lordosis (0–6%).

### 3.2. Differential Expression Analysis

Normality tests revealed that none of the gene expression values followed a normal distribution thus, non-parametric tests were selected for the rest of the analysis. To prioritize expression variables for exploratory model development, Wilcoxon rank-sum tests were used to identify nominal univariate differences between the GOOD and POOR groups at each developmental stage (Table 1). Eleven genes showed no nominally significant expression differences between the two SBS-based quality groups at any developmental stage and were therefore excluded from subsequent analyses. For the remaining genes, the nominal expression differences between the SBS-based quality groups varied among developmental stages and, in most cases, were stage-specific (Table 1). The genes showing nominally significant expression differences between the groups at three out of four stages were *uqcr11b*, *uqcrc2a*, and *wap65*. Mid-metamorphosis had the largest number of genes, showing nominally significant differences. After stage-specific Benjamini–Hochberg correction, *trf* remained significant at first feeding, whereas *uqcrc2a*, *trf*, *ebp*, *mylpfb*, *uqcr11b*, and *myl1* remained significant at mid-metamorphosis. No expression variables remained significant after FDR correction at flexion or post-flexion. The complete raw and adjusted *p*-values are provided in Table S1.

### 3.3. Correlation and Multivariate Analysis

**Figure A1:**
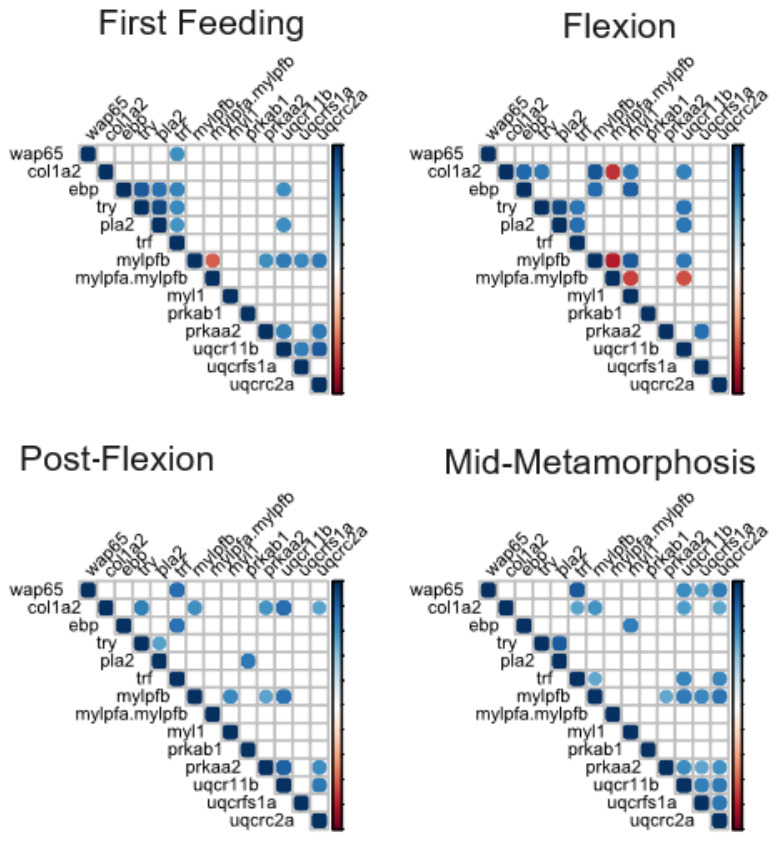
Correlation analysis between genes for each developmental stage.

Correlation analysis revealed the genes that were highly correlated (Figure A1) for each of the four developmental stages under examination. Because collinearity can affect the final step of the analysis, particularly the random forest feature-importance estimates, one gene from each highly correlated pair was discarded when the correlation coefficient exceeded 0.8. This threshold was used as a practical criterion to reduce redundancy among predictors for model development; exclusion of a highly correlated gene does not imply that it lacks biological relevance. The gene with the lowest Wilcoxon rank-sum test *p*-value between the two SBS-based quality groups was retained, resulting in different gene sets for the four developmental stages (Table 2).

**Figure 3:**
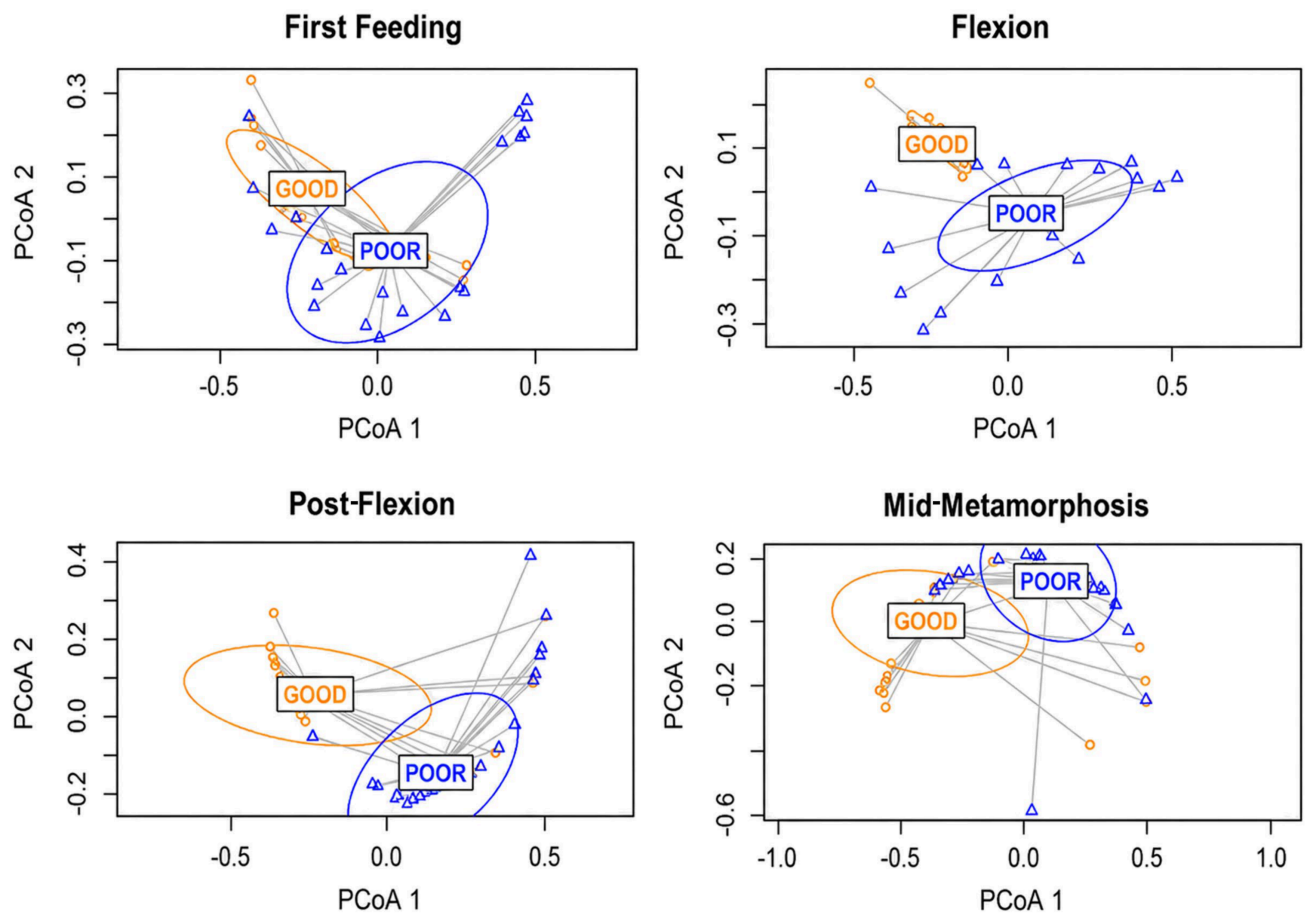
Principal Coordinate Analysis (PCoA) per developmental stage.

Multivariate analysis showed that dispersion of the data inside the groupings was similar for all developmental stages (Figure 3; Table 3), indicating that the assumptions for PERMANOVA were met in all cases. PERMANOVA indicated significant differences between the GOOD and POOR groups, although group classification explained a modest proportion of the total multivariate variation (Figure 3; Table 3). The proportion of variation explained was highest at mid-metamorphosis (22.4%), followed by first feeding and post-flexion (19.5%) and flexion (17.2%). The remaining residual variation may reflect a combination of unmeasured environmental, population-specific, biological, and technical factors, whose individual contributions could not be separated in the present study.

### 3.4. Classification (Random Forest)

**Figure 4:**
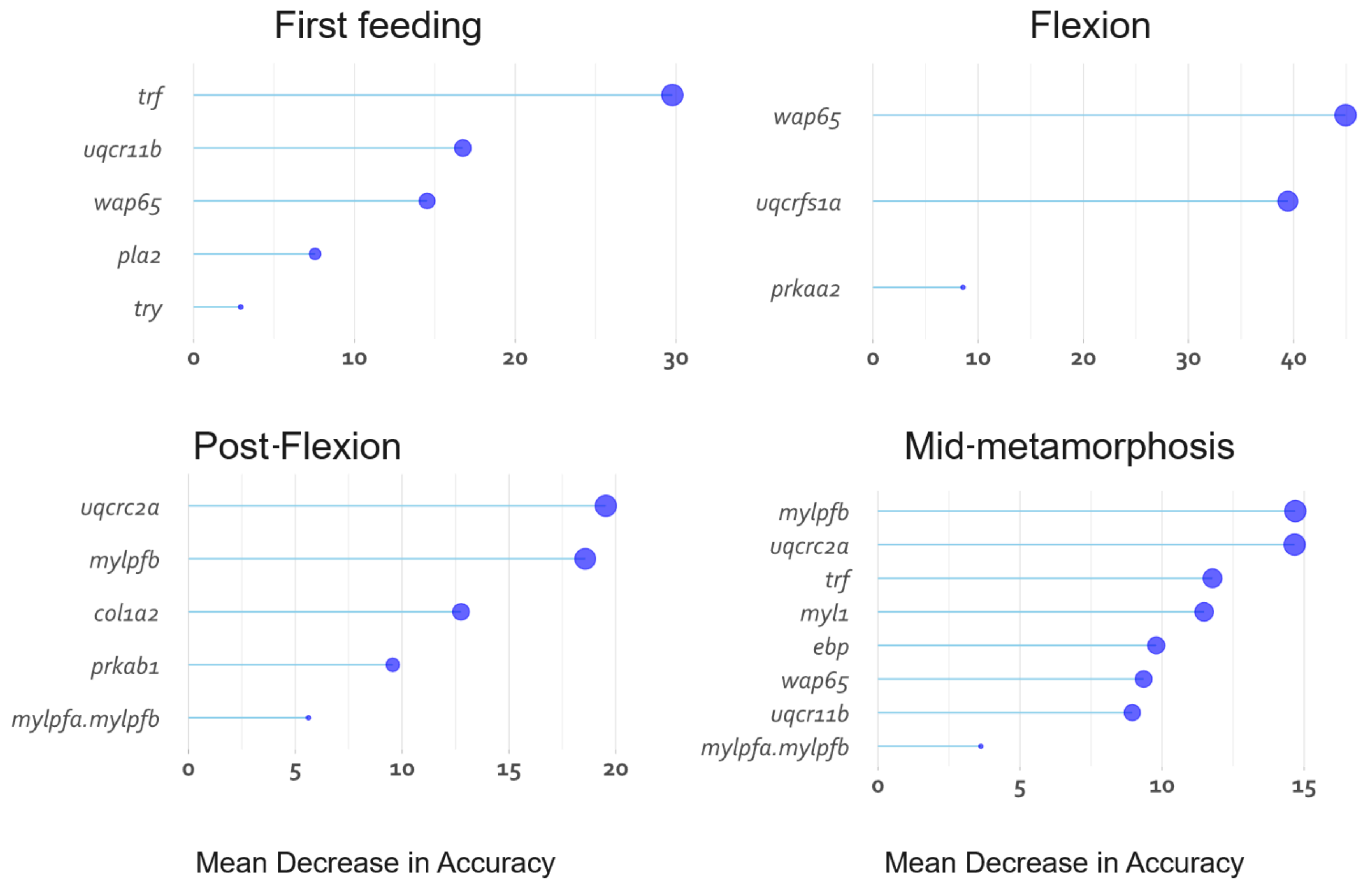
Variable importance for classification models per developmental stage. Circle size is proportional to Mean Decrease in Accuracy, with larger circles indicating greater variable importance.

Random forest classification models were developed to distinguish between “GOOD” and “POOR” groups across four developmental stages. For each stage, two model versions were trained: one using all selected variables for the stage (Table 2), and another using only the top three most informative predictors based on variable importance scores (Figure 4). Among the repeatedly selected predictors, *uqcr11b* and *uqcrc2a* are involved in mitochondrial oxidative phosphorylation, *trf* in iron transport, and *wap65* in temperature acclimation and stress- and immune-related processes. Model performance was assessed primarily using cross-validated ROC AUC. Training-set metrics were also reported descriptively, including the No Information Rate (NIR), which represents the accuracy of a naïve classifier that assigns every sample to the most frequent class. The associated one-sided test evaluates whether the observed model accuracy is statistically greater than this baseline (Table 4, Table 5, Table 6 and Table 7). Across all stages, the models yielded cross-validated area under the receiver operating characteristic curve (ROC AUC) values that ranged from 0.83 to 0.962 when trained on all variables, and 0.831 to 0.885 when using only the top three predictors. These cross-validated AUC values represent internal model performance rather than independent predictive performance and may be optimistically biased because feature selection preceded cross-validation. Cross-validation was performed at the RNA-sample level rather than by larval population, so the estimates may also partly reflect within-population similarities. The reduced-predictor models showed performance values broadly comparable to those of the corresponding full models at first feeding and post-flexion, while a small decrease in cross-validated ROC AUC was observed at mid-metamorphosis.

Cross-validated sensitivity and specificity were moderate to high across all stages, typically ranging from 0.69 to 0.78 for sensitivity and 0.67 to 0.87 for specificity when using all variables. In several instances, the reduced models showed numerically higher sensitivity or specificity. By contrast, training-set metrics were often near-perfect (accuracy and sensitivity = 1.0), illustrating the optimistic nature of evaluating a model on the same data used for model fitting and indicating possible overfitting. For this reason, greater emphasis is placed on the cross-validated metrics, which provide a more informative internal estimate of model performance than the metrics calculated on the same data used for model fitting, while the No Information Rate (NIR) offers a useful baseline for interpreting the value added by the models. The flexion-stage model trained on all variables achieved the highest cross-validated AUC (0.962), though no reduced-variable model was tested at this stage. At the mid-metamorphosis stage, the full and reduced models yielded cross-validated AUCs of 0.91 and 0.885, respectively.

## 4. Discussion

Machine learning algorithms are increasingly being applied to gene expression data, proving effective in predicting clinical and phenotypic outcomes and revealing complex biological patterns in several fields, including cancer research [47,48], personalized medicine [49], and agriculture [50,51]. In aquaculture, these approaches are also being adopted for applications such as disease detection and management [34,52], growth performance [53], and environmental responses [54]. In particular, random forest has shown competitive results compared to other machine learning algorithms and high computational efficiency for predicting disease resistance in fish using genotype data [34]. Random forest demonstrated high predictive accuracy in classifying liver and muscle metabolomic profiles into growth groups, with its sensitivity analysis identifying key metabolites for further exploration [53]. Furthermore, random forest was utilized to develop a highly accurate model for predicting the growth rate of gilthead sea bream by integrating both molecular (gene expression) and biochemical markers, enabling the early identification of faster and slower growing fish under different dietary regimes [55]. Artificial intelligence is already utilized for the detection of skeletal deformities as well but is mainly focused on phenotypic identification based on image analysis [32,36,56,57].

To our knowledge, this is the first study to apply supervised machine learning classification, specifically random forest, to gene expression data to discriminate larval populations with contrasting SBS incidence. Moreover, this represents the first effort using molecular-level data to explore saddleback syndrome (SBS). The purpose of this study was to classify European seabass larval batches according to SBS-associated quality using gene expression data and machine learning in a heterogeneous sample collection. The collected samples are representative of different genetic backgrounds, rearing conditions, and handling processes, reflecting each hatchery’s methodology for best practices. The selection of samples aimed to represent diverse rearing methodologies and fish genetic backgrounds that characterize the sector, with the goal of producing models capable of generalizing beyond variations arising from these sources and with potential for broader application. We assessed the expression of genes involved in metabolism, growth, bone formation, and energy production. Different genes were selected for each developmental stage based on significant expression differences between the GOOD and POOR groups and low collinearity among the selected genes. This strategy ensured that the respective models featured the genes that were most relevant at each developmental stage.

The ontogenetic timing of SBS development is critical for interpreting the present gene expression results. Previous work in European seabass demonstrated that SBS is not initiated at metamorphosis but rather is ontogenetically associated with severe abnormalities of the primordial marginal finfold during the flexion stage, at approximately 8.5–11.0 mm SL, including epidermal erosion and subsequent disruption of fin development [21]. This is consistent with the broader view that larval skeletal abnormalities arise during narrow developmental windows, when morphogenetic processes are particularly sensitive to environmental, nutritional, physiological, or genetic perturbations [4]. Therefore, the biological meaning of the molecular signatures identified here should be considered stage-dependent rather than uniform across ontogeny. Gene expression differences detected at first feeding precede the anatomical onset of SBS and may be consistent with molecular states associated with early susceptibility or SBS risk, potentially involving differences in the stress response, iron/redox balance, mitochondrial activity, or metabolic status of the larvae. By contrast, the flexion stage has stronger biological relevance as an early assessment window because it coincides with the reported emergence of primordial marginal finfold abnormalities and with the beginning of dorsal, anal, and pelvic fin patterning [21]. The high discriminatory performance of the flexion-stage model therefore suggests that gene expression differences at this stage coincide with the developmental window in which pathogenic processes associated with SBS may be occurring. In contrast, expression differences observed at the post-flexion, and particularly at the mid-metamorphosis stages, should be interpreted more cautiously: at these stages, SBS is likely already developing or phenotypically established, and the associated transcriptional profiles may reflect progression, tissue remodeling, altered muscle and extracellular matrix development, or physiological compensation rather than initiating causes. This distinction is important because the identified transcriptional signatures should be interpreted in relation to the developmental stage rather than as equivalent markers across ontogeny. Within the present dataset, the flexion-stage profiles showed the strongest discrimination and coincided with the reported developmental window of SBS onset.

Across the four developmental stages, the random forest models showed encouraging internal cross-validation performance, with ROC AUC values ranging from 0.83 to 0.962. The flexion-stage model achieved the highest cross-validated AUC, sensitivity, and specificity, indicating the strongest discrimination within the present dataset. The models constructed for the mid-metamorphosis and first feeding stages also showed useful internal discrimination. Reduced-variable models generally retained comparable cross-validation performance, suggesting that a smaller set of candidate genes may contain discriminatory information. However, the near-perfect fitted-model performance obtained on the training data is optimistic and should not be interpreted as evidence of generalizable predictive accuracy. Random forest variable importance scores indicate the contribution of predictors to classification within the present dataset and should not be interpreted as evidence of biological causality; the observed patterns may also reflect general developmental variations, environmental or hatchery conditions, and population-specific effects. The top predictive variables distinguishing larvae groups varied across developmental stages but consistently involved genes related to oxidative metabolism, structural development, and mineral transport. At the first feeding (FF) stage, the key predictive markers were *trf* (transferrin), *uqcr11b* (OXPHOS gene), and *wap65* (warm-temperature-acclimation-related protein). Transferrin, an iron transport protein, may influence early mineral availability and oxidative balance [58], both of which are essential during the rapid tissue differentiation occurring in early larval stages. Iron dysregulation is known to contribute to oxidative stress, which has been implicated in developmental anomalies, including skeletal deformities [59]. The gene *uqcr11b* encodes a subunit of the mitochondrial ubiquinol-cytochrome c reductase complex (Complex III). Oxidative phosphorylation (OXPHOS) is the main mechanism of cellular energy production and is intimately linked to cellular redox balance. Both energy production and redox balance are essential for supporting the cellular processes involved in tissue growth and differentiation, including bone development [60]. *wap65*, an acute-phase hemopexin-like protein, reflects early activation of stress response pathways triggered by environmental or physiological stressors. It participates in the inflammatory response and stress adaptation and plays a role in iron metabolism [61].

In the flexion (FL) stage, *wap65*, *uqcrfs1* (OXPHOS gene), and *prkaa2* (AMPK pathway) were the most predictive. The gene *prkaa2* encodes the catalytic alpha-2 subunit of AMP-activated protein kinase (AMPK), a critical cellular energy sensor that regulates metabolism [62]. It is also involved in skeletal muscle development and growth [63,64].

During the post-flexion (PF) stage, the top variables included *uqcrc2a* (an OXPHOS gene), *mylpfb* (myosin light chain), and *col1a2* (collagen type I alpha 2). The latter two are directly involved in muscle development and growth and skeletal matrix composition, respectively. The *mylpfb* gene (myosin light chain, phosphorylatable, fast skeletal muscle b) encodes a myosin light chain protein. It plays a key role in muscle fiber morphogenesis and skeletal muscle development through its involvement in myofibril assembly and hyperplastic muscle growth [65]. *col1a2* is involved in forming the scaffold of the bone extracellular matrix, providing the structural support that is essential for proper bone mineralization, thus shaping bone morphology [66,67], while abnormal collagen formation is associated with pathological conditions [68]. At this stage, the most informative variables shifted toward genes related to muscle development and extracellular matrix composition.

In the metamorphosis (MM) stage, *trf*, *mylpfb*, and *uqcrc2a* were the most informative. The recurrence of *trf* from FF to MM may indicate that iron homeostasis and redox balance remain critical throughout development. The genes *mylpfb* and *uqcrc2a*, also important at PF, reinforce the importance of muscle integrity and mitochondrial energy production in this high-demand transition to the juvenile form.

The random forest models showed encouraging internal cross-validation performance across larval developmental stages; however, several limitations should be considered. Although the total number of RNA samples was 142, these samples originated from only eight larval populations, and dividing the dataset by developmental stage and SBS-incidence group reduced the number of samples available for each model. This limited population-level sample size may increase the likelihood of model overfitting and restrict the generalizability of the results to new populations. Repeated 10-fold cross-validation was used to estimate model performance, while SMOTE was applied during model training to address class imbalance. Because feature selection was performed on the complete stage-specific datasets before cross-validation, information from the validation folds contributed to predictor selection. This represents a source of information leakage and may have led to optimistic estimates of cross-validated model performance. In addition, because cross-validation folds were generated at the individual RNA-sample level rather than grouped by larval population, RNA samples originating from the same population could occur in both training and validation folds. This lack of population-level independence may have further contributed to optimistic estimates of cross-validated performance. Nevertheless, these procedures do not replace validation using an independent population cohort.

These observations support the biological plausibility of the identified expression patterns; however, they do not exclude model overfitting, population-specific effects, or influences arising from hatchery conditions. Unmeasured parental effects, genetic background, rearing conditions, and other environmental variables may also have contributed to the transcriptional differences independently of SBS incidence, and their individual effects could not be separated in the present study. The results should therefore be interpreted as exploratory evidence of batch-level discrimination rather than validation of a predictive or diagnostic tool. The Wilcoxon tests were used as an exploratory feature-screening procedure rather than as confirmatory differential expression analysis. Although several variables showed nominal univariate differences between SBS-incidence groups, only one subset remained significant after stage-specific FDR correction. Variables that did not remain FDR significant may still contribute jointly to multivariable classification, but their individual associations should be interpreted as exploratory rather than as independently confirmed differential expression markers. A further limitation is that the gene expression results were not validated at the protein level. Changes in transcript abundance do not necessarily result in corresponding changes in protein abundance or function, particularly for structural candidates such as collagen- and myosin-related genes. Therefore, these genes should be regarded as candidate expression markers associated with SBS incidence rather than validated functional biomarkers. Future studies should combine transcript measurements with protein-level assays and functional analyses to assess their biological and practical marker potential.

Another limitation that should be acknowledged relates to the nature of the samples used for gene expression analysis. Due to technical constraints, the same individual larvae that were phenotypically characterized using SBS could not be used for RNA extraction and gene expression profiling. This was because the staining process used for skeletal evaluation irreversibly degrades RNA. As a result, expression analysis had to be performed on larvae from the same population but not on the exact individuals assessed for SBS. Consequently, the models evaluate associations with population-level SBS incidence and cannot be interpreted as predicting the SBS status of individual larvae.

As a logical next step, future studies should consider coupling real-time, non-destructive skeletal evaluations such as microscopy methods that preserve RNA integrity, with immediate sampling for gene expression analysis. This approach would enable the direct correlation of phenotypic outcomes with transcriptional profiles from the same individuals, improving the accuracy of biological interpretation and potentially identifying causal mechanisms with greater precision. Expanding the gene panel and employing high-throughput sequencing could help capture the additional biological processes involved in larval development. Future larger-scale studies integrating gene expression profiles with family structures and genomic variations could determine whether SBS-associated expression patterns are linked to heritable factors that may ultimately inform marker-assisted selective breeding. Lastly, while the present study was not designed to identify causal mechanisms, it needs to be stated that the observed gene expression patterns likely reflect broader physiological responses associated with SBS rather than their direct causes.

## 5. Conclusions

Overall, these results support the potential of gene expression profiling combined with supervised machine learning as an exploratory approach for stage-specific discrimination of European seabass larval populations with contrasting SBS incidence. The identified transcriptional signatures should not be considered equivalent across developmental stages and may represent stage-dependent transcriptional patterns associated with larval populations exhibiting contrasting SBS incidence. These findings suggest that the flexion stage represents a promising candidate window for early SBS-related molecular assessment. Further validation using larger, independent populations and sampling that links molecular profiles with individual phenotypes will be required before the identified stage-specific molecular signatures can be considered suitable for commercial hatchery application.

## Figures and Tables

**Table 1:** Nominal Wilcoxon rank-sum test results used for exploratory feature screening at each developmental stage. FF, first feeding; FL, flexion; PF, post-flexion; MM, mid-metamorphosis. * = *p* < 0.05; ** = *p* < 0.01; *** = *p* < 0.001.

Gene Code	Gene	Function	FF	FL	PF	MM
*col1a2*	Collagen 1α2	Bone formation	-	-	*	-
*col10a1*	Collagen type X alpha 1 chain	Bone formation	-	-	-	-
*try*	Trypsin	Digestion	*	-	-	-
*prkab1*	5′-AMP-activated protein kinase subunit beta-1	Energy metabolism	-	-	*	-
*prkag2_L*	AMP-activated, gamma 2 non-catalytic subunit L homeolog	Energy metabolism	-	-	-	-
*prkaa2*	Protein kinase AMP-activated catalytic subunit alpha 2	Energy metabolism	-	*	-	-
*prkag3*	5′-AMP-activated protein kinase subunit gamma-3	Energy metabolism	-	-	-	-
*aakb1*	Protein kinase AMP-activated non-catalytic subunit beta 1	Energy metabolism	-	-	-	-
*fgb*	Fibrinogen beta chain	Extracellular matrix component	-	-	-	-
*apoa1*	Apolipoprotein A1	Lipid metabolism	-	-	-	-
*ebp*	3-beta-hydroxysteroid-delta(8)	Lipid metabolism	*	-	-	**
*pla2*	High unsaturated fatty acids (HUFA)-phospholipase A2	Lipid metabolism	*	-	-	-
*trf*	Transferrin	Iron transport and homeostasis	***	-	-	***
*mylpfa*	Myosin light chain, phosphorylatable, fast skeletal muscle a	Myogenesis	-	-	-	-
*mylpfb*	Myosin light chain, phosphorylatable, fast skeletal muscle b	Myogenesis	-	-	*	**
*mylpfa/mylpfb*	Mylpfa/mylpfb ratio	Myogenesis	-	-	*	*
*myl1*	Myosin light chain 1	Myogenesis	-	-	-	**
*mylz3*	Myosin, light polypeptide 3, skeletal muscle	Myogenesis	-	-	-	-
*uqcr11a*	OXPHOS CIII subunit	Oxidative phosphorylation	-	-	-	-
*uqcr11b*	OXPHOS CIII subunit	Oxidative phosphorylation	**	-	*	**
*uqcrfs1a*	OXPHOS CIII subunit	Oxidative phosphorylation	-	*	-	-
*uqcrfs1b*	OXPHOS CIII subunit	Oxidative phosphorylation	-	-	-	-
*uqcrc2a*	OXPHOS CIII subunit	Oxidative phosphorylation	*	-	**	***
*ndufs1a*	OXPHOS CI subunit	Oxidative phosphorylation	-	-	-	-
*wap65*	Warm-temperature-acclimation-related-65 kDa-protein or hemopexin	Temperature acclimation, stress, and immune response	*	*	-	*

**Table 2:** Genes used as input for the classification analysis for each of the developmental stages. FF, first feeding; FL, flexion; PF, post-flexion; MM, mid-metamorphosis.

FF	FL	PF	MM
*wap65*	*wap65*	*col1a2*	*wap65*
*try*	*prkaa2*	*mylpfb*	*ebp*
*pla2*	*uqcrfs1a*	*mylpfa/mylpfb*	*trf*
*trf*		*prkab1*	*mylpfb*
*uqcr11b*		*uqcrc2a*	*mylpfa/mylpfb*
			*myl1*
			*uqcr11b*
			*uqcrc2a*

**Table 3:** Statistical significance for dispersion and PERMANOVA analysis per stage; R2 represents the variability explained by the groupings (GOOD, POOR). FF, first feeding; FL, flexion; PF, post-flexion; MM, mid-metamorphosis.

Stage	Dispersion *p*-Value	PERMANOVA *p*-Value	R^2^
FF	0.489	0.002	0.195
FL	0.107	0.043	0.172
PF	0.361	0.001	0.195
MM	0.537	0.002	0.224

**Table 4:** Random forest classification model metrics for first feeding stage. For each metric, left represents the model trained with all selected stage variables and right only with the top three most informative variables.

	Cross-Validated Performance (Model)	Fitted Model Performance (Training Data)
ROC (AUC)	0.835/0.853	0.984/0.98
Accuracy	—	0.969/0.99
Sensitivity	0.72/0.75	1/1
Specificity	0.763/0.793	0.952/0.857
Kappa	—	0.935/0.813
No Information Rate	—	0.636/0.636
*p*-Value [Acc > NIR]	—	6.61 × 10^−6^/4.03 × 10^−4^

**Table 5:** Random forest classification model trained with all selected flexion-stage variables.

	Cross-Validated Performance (Model)	Fitted Model Performance (Training Data)
ROC (AUC)	0.962	1
Accuracy	—	1
Sensitivity	0.775	1
Specificity	0.87	1
Kappa	—	1
No Information Rate	—	0.6923
*p*-Value [Acc > NIR]	—	7.04 × 10^−5^

**Table 6:** Random forest classification model metrics for post-flexion stage. For each metric, left represents the model trained with all selected stage variables and right only with the top three most informative variables.

	Cross-Validated Performance (Model)	Fitted Model Performance (Training Data)
ROC (AUC)	0.83/0.831	1/1
Accuracy	—	0.975/0.951
Sensitivity	0.69/0.753	0.944/0.888
Specificity	0.67/0.696	1/1
Kappa	—	0.950/0.899
No Information Rate	—	0.561/0.561
*p*-Value [Acc > NIR]	—	1.68 × 10^−9^/2.73 × 10^−8^

**Table 7:** Random forest classification model metrics for the mid-metamorphosis stage. For each metric, left represents the model trained with all selected stage variables and right only with the top three most informative variables.

	Cross-Validated Performance (Model)	Fitted Model Performance (Training Data)
ROC (AUC)	0.91/0.885	1/1
Accuracy	—	1/1
Sensitivity	0.783/0.753	1/1
Specificity	0.75/0.72	1/1
Kappa	—	1/1
No Information Rate	—	0.523/0.523
*p*-Value [Acc > NIR]	—	1.60 × 10^−12^/1.60 × 10^−12^

## Data Availability

The original contributions presented in this study are included in the article. Further inquiries can be directed to the corresponding author.

## Supplementary Materials

The following supporting information can be downloaded at: https://www.mdpi.com/article/10.3390/ani16152375/s1. Table S1. Stage-specific Wilcoxon and FDR.

## Appendix A

**Table A1:** Sample collection characterization. Eight populations were included, four from each hatchery. The four populations from Hatchery A contributed 12, 10, 23, and 18 samples, whereas the four populations from Hatchery B contributed 21, 17, 18, and 23 samples, respectively.

Hatchery	GOOD	POOR
A	22	41
B	38	41
Total	60	82
**Developmental Stage**		
First Feeding (FF)	13	20
Flexion (FL)	8	18
Post-Flexion (PF)	17	24
Mid-Metamorphosis (MM)	22	20

**Table A2:** Primer list and reaction efficiencies.

Gene Code	Gene	Ensembl Gene ID	FW Primer	RV Primer	Efficiency	Amplicon Length (bp)
*wap65*	Warm-temperature-acclimation-related 65 kDa protein or hemopexin (*wap65*)	ENSDLAG00005021309	ATCAAACTCAATGCCTTCACACC	AGCACTCGCCCTCACTAATGG	90.3	97
*apoa1*	Apolipoprotein A1	ENSDLAG00005000348	CTGGAGAGCCTGAGAGCAATGG	TGTTGATGTTCTGAGCCTGGTTGT	115	93
*col1a2*	Collagen 1α2	CX660451	TCGCCCAGAAGAACTGGTACAGAA	CGTTGTAGGTAAACTCAGTACCACCG	100.6	93
*fgb*	Fibrinogen beta chain (*fgb*)	ENSDLAG00005002651	ACCGTCCGCTATCAAGAGG	CTTCTCCTGTGCCTGTGGTC	95	131
*ebp*	3-beta-hydroxysteroid-Delta(8) (*ebp*)	ENSDLAG00005001286	CCACCTATGTTGCCAATGACC	AACCAGCCCTCAATGACACC	90.4	196
*try*	Trypsin	ENSDLAG00005013031	CTCCCTGGTCAACGAGAACT	ACCCTGATGTTGTGCTCTCC	100.7	90
*pla2*	High unsaturated fatty acids (HUFA)-Phospholipase A2	ENSDLAG00005004463	TCCTGTGTGTGATGCCTGAT	TCTCGTCGCAGTTGTAGTCG	100.1	212
*trf*	Transferrin (*trf*)	ENSDLAG00005008061	ACACTGCTGGACTGAACAACTACGA	GGATTTCTTCCCGCTGAGGT	92.4	146
*col10a1*	Collagen Type X Alpha 1 Chain	ENSDLAG00005004562	TGGGAATGAGTGAGGTTATGG	GGATGCTGTAGGCAAAATAGT	94.7	192
*mylpfa*	myosin light chain, phosphorylatable, fast skeletal muscle a	ENSDLAG00005010562	CATGTTTGAGCAGAGCCAGA	CACGTCCCTCAGGTCATCTT	106.1	100
*mylpfb*	myosin light chain, phosphorylatable, fast skeletal muscle b	ENSDLAG00005007525	TCATCAGCAAGGACGATCTG	GATCAGCACCCTTCAGCTTC	98.7	156
*myl1*	Myosin Light Chain 1	ENSDLAG00005002632	CACCCAAGAAGGACGCTAAG	GGGCTGAACTCGATCTTGAC	96.6	136
*mylz3*	myosin, light polypeptide 3, skeletal muscle	ENSDLAG00005026275	GACTACGTTGAGGGTCTGCG	ACACTGCCGTTCTCATCCTC	102.9	155
*prkab1*	5′-AMP-activated protein kinase subunit beta-1	ENSDLAG00005004430	CTCAGTCGCTCCAATCCTAC	ATTACCGCCACTCTATGTCTAC	102.2	106
*prkag2_L*	AMP-activated, gamma 2 non-catalytic subunit L homeolog	ENSDLAG00005002525	AGGCTGGTTGTCGTTGATGAG	TGTGTCTGGTTCTGTTGAGTCC	97.4	153
*prkaa2*	Protein Kinase AMP-Activated Catalytic Subunit Alpha 2	ENSDLAG00005005389	GGACGGGTGGAAGATACAGA	CGTAGGAACTCCCCATCTGA	98.7	188
*prkag3*	5′-AMP-activated protein kinase subunit gamma-3	ENSDLAG00005017447	TCACAGGTGTCATTCCCAAA	TTTGCCACCAGTGCATAAAA	103.2	179
*aakb1*	Protein Kinase AMP-Activated Non-Catalytic Subunit Beta 1	ENSDLAG00005011830	GCTCAACAGAAGTCAGAAGAAC	CCACAAACCGTCCACACAGAA	104	88
*uqcr11a*	OXPHOS CIII	ENSDLAG00005024816	GATCCCAACTTTGGCTGTATGG	TGCCACTGATGTAGGGAACGT	99.5	101
*uqcr11b*	OXPHOS CIII	ENSDLAG00005007897	GGCGGCAAATACATTGCTA	ATCGCCAGTCTGTGAAGTGA	98.3	100
*uqcrfs1a*	OXPHOS CIII	ENSDLAG00005020221	TTGCCCACACAGACATTAGG	CTTCTGGATTCACTGCTCTCC	106.9	100
*uqcrfs1b*	OXPHOS CIII	ENSDLAG00005021271	GGCAAAAACATGACCTTCAA	GCTCCCCCATATTCACAGAC	100.5	100
*uqcrc2a*	OXPHOS CIII	ENSDLAG00005014897	AGAACTACTCCCCCGTGTCC	GCCAGTCGTAGCACATGAGA	96.3	100
*ndufs1a*	OXPHOS CI	ENSDLAG00005009594	GGCTCCTCTCTTCAATGCAC	CAACCACAACAACAGGATGC	105.1	200
*rpl13*	Ribosomal Protein L13	ENSDLAG00005000624	GAAGGCATCAACATCTCC	CTCTGAAGTGGTAAGGTC	99.1	109
*fau*	FAU Ubiquitin Like And Ribosomal Protein S30 Fusion	ENSDLAG00005000664	CTTCGTGAATGTTGTGCC	ACTGATGGATGGTGATGA	102.9	103
